# Dr. Harrison, on Opium in Syphilis

**Published:** 1810-07

**Authors:** J. E. Harrison

**Affiliations:** Thornbury


### *To the Editors of the Medical and Physical Journal.*

Gentlemen,

I Beg leave to lay before you the following singular cure of Syphilis by Opium. In the month of April, 1808, W. Vaughan, a cooper at Thornbury, Gloucestershire, applied to me, after he had been declared by all the medical men of this town, incurable of the syphilis. He, as he informs me, had been a whole year under the care of a practitioner of this place, without success; nor did any person expect him to survive. Both of the tibiæ were carious, as well as the os malæ and processus mastoidei; there was also a large tumour, about the size of a double fist, on the os frontis, apparently filled with matter, which was absorbed. He had a hectic fever, a quick, thready pulse, about 90, with excruciating pains, as he described it, of the whole frame. He was very much emaciated, and not able to walk without help. I begun the cure with a grain of opium and one of extract. cicutæ in a pill, which was repeated every six or eight hours, occasionally oftener, until the pains were abated. He had no appetite, nor did he, he assured me, know for some time past what sleep was.

was. The opium procured ease from pain in less than 24 hours, with some sleep. To the carious bones, acidum mur. on sponge tents was applied, and large poultices of bread and milk over the tents, which were well soaked in acid. muriat. and renewed twice every twenty-four hours.

In June following he was considerably relieved, and the opium was sometimes omitted during the day. In August, the shin bones were exfoliated, and the tumour on the frontal bone totally absorbed. The beginning of September he took bark and steel, with ext. cicutæ, three times a day, which were continued in a mixture till the latter end of October, when he was so much recovered as to rest well with one opium pill every night at bed-time. In November he had gained much strength and flesh, and in December he rested without opium.

January 6, 1809. After being reduced to a mere skeleton, and I may with truth say, almost entered into the vale of death, he perfectly recovered.

May 23, 1810. He remains radically cured, and in perfect health; fat, strong, and robust. I attribute this cure principally to the liberal use of opium.

His wife, and a child about nine months old, were both greatly infected. I cured them by repeated and gentle alteratives. The woman, a few months ago, has been delivered of a seven months child, free from disease. Before her delivery she had been very ill of a dysentery.

I am, &c.

J. E. HARRISON, M. D.

*Thornbury, May* 23, 1810.

